# International migration patterns of Red-throated Loons (*Gavia stellata*) from four breeding populations in Alaska

**DOI:** 10.1371/journal.pone.0189954

**Published:** 2018-01-10

**Authors:** Sarah E. McCloskey, Brian D. Uher-Koch, Joel A. Schmutz, Thomas F. Fondell

**Affiliations:** U.S. Geological Survey, Alaska Science Center, Anchorage, Alaska, United States of America; Linnaeus University, SWEDEN

## Abstract

Identifying post-breeding migration and wintering distributions of migratory birds is important for understanding factors that may drive population dynamics. Red-throated Loons (*Gavia stellata*) are widely distributed across Alaska and currently have varying population trends, including some populations with recent periods of decline. To investigate population differentiation and the location of migration pathways and wintering areas, which may inform population trend patterns, we used satellite transmitters (n = 32) to describe migration patterns of four geographically separate breeding populations of Red-throated Loons in Alaska. On average (± SD) Red-throated Loons underwent long (6,288 ± 1,825 km) fall and spring migrations predominantly along coastlines. The most northern population (Arctic Coastal Plain) migrated westward to East Asia and traveled approximately 2,000 km farther to wintering sites than the three more southerly populations (Seward Peninsula, Yukon-Kuskokwim Delta, and Copper River Delta) which migrated south along the Pacific coast of North America. These migration paths are consistent with the hypothesis that Red-throated Loons from the Arctic Coastal Plain are exposed to contaminants in East Asia. The three more southerly breeding populations demonstrated a chain migration pattern in which the more northerly breeding populations generally wintered in more northerly latitudes. Collectively, the migration paths observed in this study demonstrate that some geographically distinct breeding populations overlap in wintering distribution while others use highly different wintering areas. Red-throated Loon population trends in Alaska may therefore be driven by a wide range of effects throughout the annual cycle.

## Introduction

Until recently seabird migration patterns have been poorly understood. This knowledge gap is partly due to the past reliance on re-sighting and recovery data of banded birds to estimate movement, which limits information about actual migration paths, speeds, and stopover locations [1–2]. Recent developments in light-based geolocators and satellite transmitter technology now allow researchers to track individuals throughout breeding and non-breeding periods, both near and offshore, thus facilitating a better understanding of migratory patterns [3–5]. Despite new technology, there has been little synthesis of seabird migration patterns as they exhibit a diversity of migration patterns [6–9]. Consequently, we have little guidance for predicting migration behavior of unstudied species and genera. Information on migration and non-breeding distribution is important because anthropogenic effects and ecological processes during these times impact mortality and population abundance [10].

Red-throated Loons (*Gavia stellata*) are migratory seabirds that breed at high latitudes, nest in low-densities on small ponds in coastal tundra ecosystems, and spend the majority of the remaining year (~ 8 months) on coastal marine waters [11]. Similar to many seabird species, little information exists regarding the migratory patterns of loons, with only a few studies examining migration of Common Loons (*G*. *immer*) in the United States south of 50 degrees of latitude [12–15] and preliminary information from Yellow-billed Loons (*G*. *adamsii*) in Alaska [16–17]. Within North America, general migration patterns of Red-throated Loons have been assessed using re-sighting and point counts [18–19]. However, such data are limited to temporal patterns in Atlantic and inland regions and lack a large scale geographic scope of migration. Throughout their circumpolar distribution, migration patterns and wintering distributions of Red-throated Loons have never been quantified and published.

A variety of threats can impact Red-throated Loon populations during migration and winter and come from both natural [20] and anthropogenic sources [21]. These threats include contaminant exposure [21], oil spills [22–23], eutrophication [24–25], bycatch in fishing operations [23, 26–27], wind farms [28–29], or habitat loss or alteration [30–31]. Given that Red-throated Loons rely heavily on marine ecosystems throughout the annual cycle and that these ecosystems may be changing rapidly due to climate change [32], understanding their migratory pathways and wintering areas may help managers determine appropriate areas and management strategies to focus on for reducing impacts that may be driving loon population declines.

Red-throated Loon breeding populations in Alaska experienced a 53% decline from 1977 to 1993 [33]. The decline was most significant in the Yukon-Kuskokwim Delta and Seward Peninsula regions [33]. Recent data suggest that Red-throated Loon populations in northern Alaska have continued to decline [34], while populations elsewhere in the state have stabilized [35]. Since data about where Red-throated Loon breeding populations migrate and spend the winter are lacking, we are hampered in our ability to identify the geographic scale and nature of potential drivers on population trends as observed in Alaska.

In this study we use satellite telemetry to identify migratory patterns of four geographically distinct breeding populations of Red-throated Loons in Alaska: the Arctic Coastal Plain (ACP), Seward Peninsula (SP), Yukon-Kuskokwim Delta (YKD) and Copper River Delta (CRD). These populations are distributed across 10° of latitude and represent the populations with greatest abundance or density of this species throughout Alaska [33]. Our objectives were to contrast the annual geographic scope and timing of migration among the four populations, and determine whether multiple breeding populations share migration and wintering habitats.

## Materials and methods

### Capture and transmitter deployment

**Fig 1:**
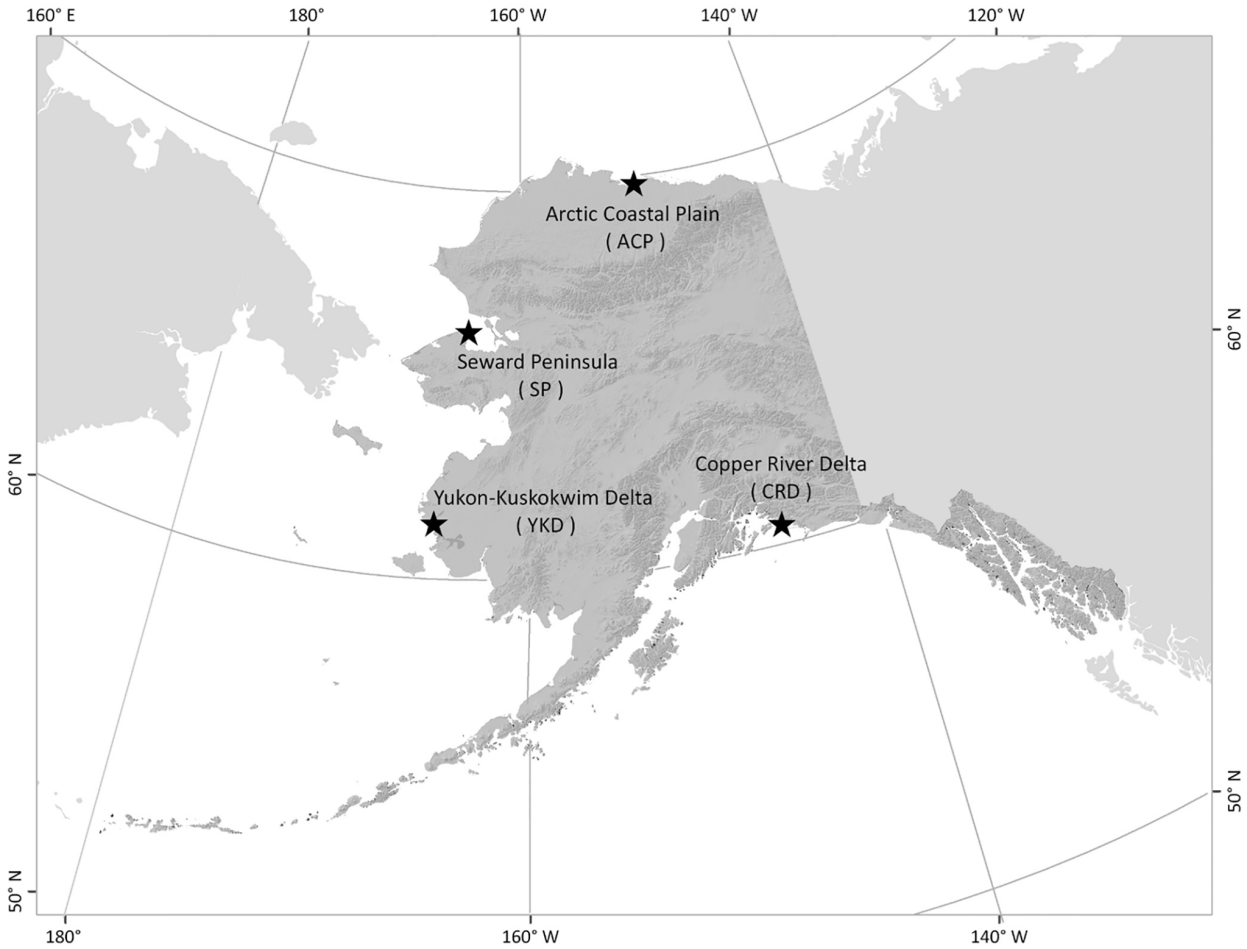
Locations of four geographically distinct breeding populations of Red-throated Loons in Alaska. Satellite transmitters were deployed on Red-throated Loons at these locations between 2000 and 2010. The Copper River Delta is in an open boreal forest whereas the other study sites were in treeless tundra.

Between 2000 and 2010, we captured and marked breeding Red-throated Loons (n = 38) with satellite transmitters at four sample regions of Alaska (Fig 1). We captured individuals using bow-net nest traps [36] during incubation or a dive net when adults were tending broods [37]. At the field site, an experienced veterinarian surgically implanted satellite transmitters, i.e., platform terminal transmitters (PTTs), into the abdominal cavity with an antenna protruding out of the body [38–39]. Each PTT weighed approximately 40 g (Microwave Telemetry, Inc., Columbia, MD). All capture and surgical procedures were in accordance with U.S. Geological Survey (USGS) Standard Operating Procedures [38–39] and approved by the USGS Animal Care and Use Committee. Any Red-throated Loons that died at the study area within two weeks of surgery were censored from the analysis (n = 5), attributing such mortality to the surgical process [38].

We programmed transmitters to elicit signals for 8 hours followed by a quiescent period of 48 to 120 hours, depending on season and year of study. We used the CLS Argos system (CLS America, Lanham, Maryland) to collect location data and filtered all data using the Douglas Argos-Filter Algorithm [40] to exclude improbable locations based on user thresholds. Locations of high quality class (L1, L2 and L3) were always retained, whereas retention of locations of class L0, LA and LB were assessed by the Douglas Argos-Filter Algorithm. For our analysis, we applied a threshold of 110 km/hr maximum sustainable rate for the ‘distance angle rate’ filter and 10 km distance threshold for ‘maximum redundant distance’ filter.

Many individuals (*n* = 9) experienced gaps in transmitter signals beyond that of the scheduled duty cycle, lasting days to months. In particular, after 2005, loons that traveled west of Hokkaido, Japan, were no longer detected until their easterly return in early spring. An unknown source of signal interference impeded the acquisition of Argos locations; a similar zone of signal interference exists in Western Europe (Microwave Telemetry, personal communication). Thus, portions of some specific migration paths near the winter terminus are unknown and arrival and departure dates may be biased. For an overview of each Red-throated Loon that was implanted with a PTT that includes sex, deployment dates, end dates, and fates, see supporting information (S1 Table).

### Analysis

We defined the beginning of fall migration by an individual loon as the date the loon left the breeding area or adjacent marine waters. Because stopover and wintering sites varied spatially and temporally between individuals and among years, we chose not to define stopover and wintering sites by specific date ranges or distance constraints, but instead defined such sites as locations where a given loon did not progress in the general migration direction for more than a week. Some loons moved from their nest lake to adjacent marine waters following nest failure but we did not consider these as stopovers. Generally, we defined fall stopover as August–November, wintering area November—April and spring stopover as April–June. We defined the end of spring migration as the date the loon returned to the breeding area and no further movements were included in the analysis. PTTs were deployed over six separate years so ordinal date (month and day only) was used as the temporal variable allowing comparisons to be made across years.

We calculated distances to fall stopover and wintering sites by summing the lengths of vectors created from point to point PTT transmissions between first day of departure from the breeding grounds to the first day at stopover or wintering site. Duration of migration was the number of days between first day of departure from the breeding grounds to first day at stopover or wintering site. To calculate the pace of migration, we divided migration distance by migration duration.

We used Geographic Information Systems (ArcGIS v.10.1) and Geospatial Modeling Environment (GME v.0.7.2.0) [41] to quantify migration movements and ArcGIS to plot locations, calculate distances (azimuthal equidistant projection) and inspect migratory patterns. We used GME to create migration paths by generating straight-line vectors between PTT locations.

## Results

### Transmitters

We used data from 32 PTTs among four separate breeding populations from 2000–2010, including 14 from the ACP, 6 from the SP, 7 from the YKD, and 5 from the CRD (Table 1). We obtained a total of 19,943 Argos locations, 63.8% were of high quality (class L1, L2 or L3), 35.9% of low quality (class L0, LA or LB), and 0.3% were of class Z and were eliminated from the analysis. Ten of the 32 transmitters lasted a full annual cycle, including migration back to the initial breeding area.

**Table 1:** The number of satellite transmitters (PTTs) deployed for each Red-throated Loon breeding population by year.

		Deployment Year					
Population^a^	Total	2000	2001	2002	2008	2009	2010
ACP	17 (3)	5 (1)		2 (1)	3 (0)	3 (1)	4 (0)
SP	7 (1)		7 (1)				
YKD	7 (0)	5 (0)	2 (0)				
CRD	7 (2)	4 (1)	3 (1)				

Numbers in parenthesis represent those transmitters that were not included in the analyses that failed prematurely or were bird mortalities before leaving the breeding grounds.

^a^ ACP, SP, YKD, and CRD represent the Arctic Coastal Plain, Seward Peninsula, Yukon-Kuskowkim Delta, and Copper River Delta breeding populations.

### General migration paths

**Fig 2:**
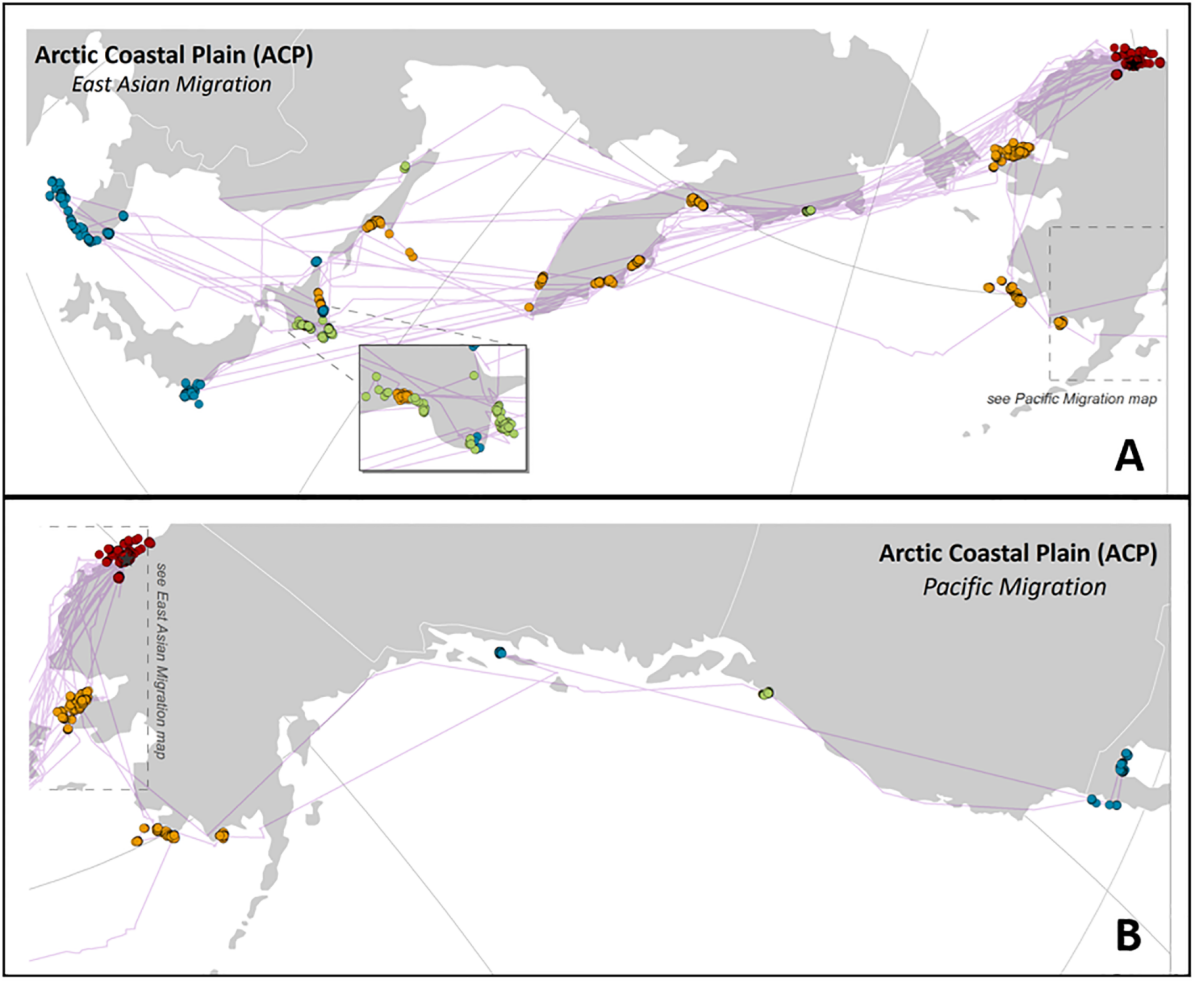
Migration patterns, stopover sites and wintering areas for Red-throated Loons breeding on the Arctic Coastal Plain (ACP) in northern Alaska. A) East Asian migration of Red-throated Loons. B) Pacific migration of a single Red-throated Loon from the Arctic Coastal Plain breeding area. Red dots represent breeding area locations, yellow dots are fall stopover sites, blue dots are wintering area locations, and green dots are spring stopover sites.

For individual specific fall/spring stopover and wintering site information see supporting information (S2 Table). Red-throated Loons marked in the ACP were the only birds to migrate westward to East Asia. Thirteen of 14 ACP loons traveled through the Bering Strait or flew over the Chukotka Peninsula, and then migrated along the coast of the Kamchatka Peninsula, and across the Sea of Okhotsk to overwintering locations along the eastern coast Japan and southern coast of South Korea (Fig 2A). The remaining loon from the ACP migrated along the eastern Pacific coast and wintered along the Baja Peninsula in Mexico (Fig 2B). ACP loons had the lowest wintering latitude of all four populations (36°26’N, SD ± 3°50’). The return migration in spring was very similar geographically but quicker in pace (see below).

**Fig 3:**
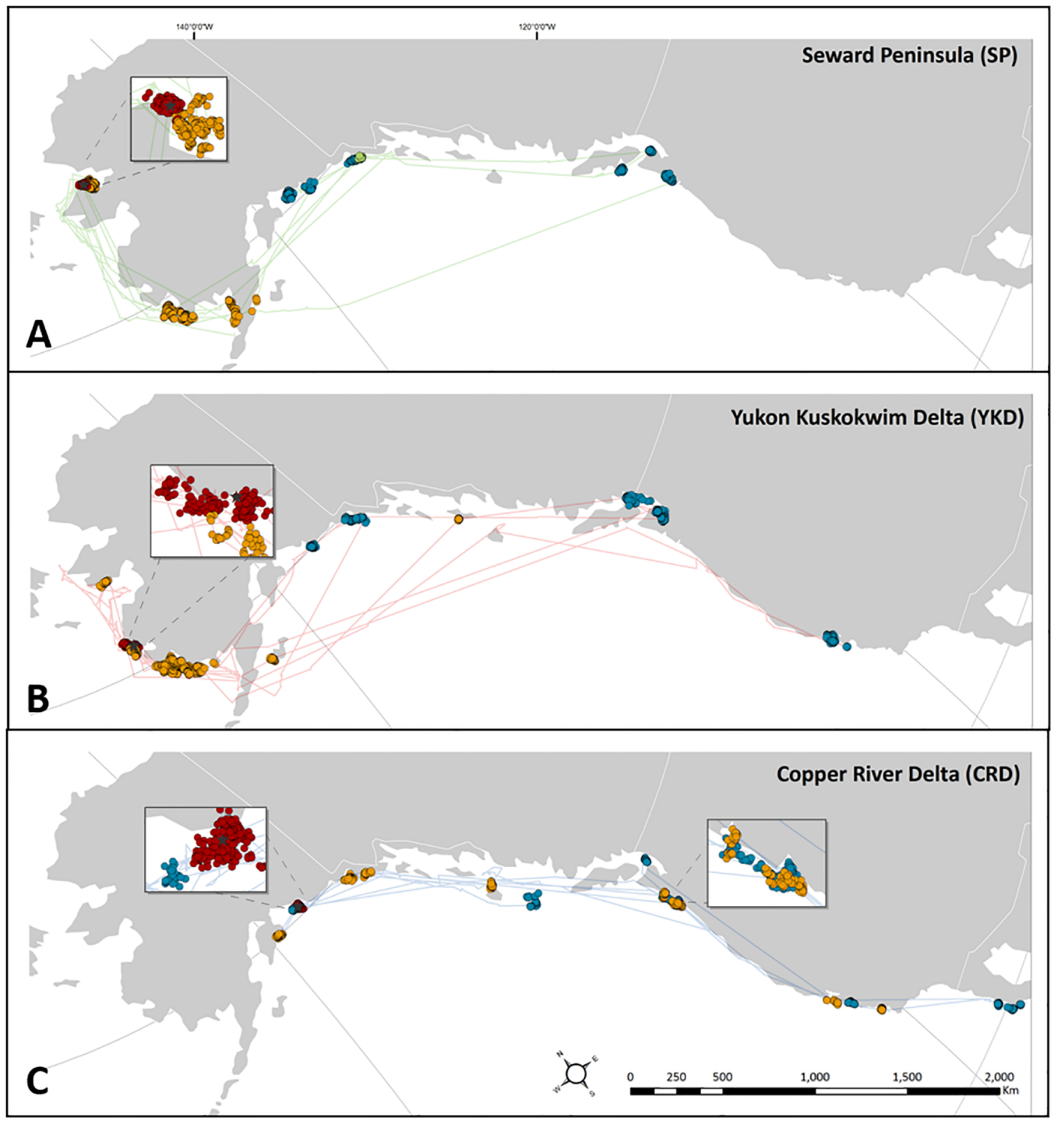
Migration patterns, stopover sites, and wintering areas of Red-throated Loons from 3 breeding populations. Red-throated Loon breeding areas in Alaska include the A) Seward Peninsula (SP), B) Yukon-Kuskokwim Delta (YKD), or C) Copper River Delta (CRD). Red dots represent breeding area locations, yellow dots are fall stopover sites, blue dots are wintering area locations, and green dots are spring stopover sites.

All six SP loons migrated southeast along the Alaska and Pacific coasts (Fig 3A). Two transmitters failed early in fall migration, two loons overwintered in southern Alaska, and the remaining two wintered within the Salish Sea near the United States and Canada border. We detected a spring migration for one SP loon which followed its fall migration route back to the western Alaska breeding area.

Six loons breeding on the YKD migrated along the eastern Pacific coast (Fig 3B). These individuals followed similar general migration paths as SP birds. One loon remained within Alaska and overwintered near Yakutat, AK. Three loons wintered in the Salish Sea, and two continued south along the Pacific coast and overwintered near San Francisco Bay, CA. All YKD transmitters failed before spring.

Two loons from the CRD had similar wintering areas as SP and YKD near the Salish Sea and San Francisco Bay area (Fig 3C). The final three CRD loons had similar wintering sites as the single ACP bird in northern Baja California, Mexico.

The mean wintering latitude of the three populations wintering in the eastern Pacific were 55°41’N (SD ± 7°34’) for SP, 46°04’N (SD ± 10°25’) for YKD, and 41°11’N (SD ± 3°50’) for CRD.

### Stopover locations

Three loons from the ACP stayed within Alaska for fall stopover. Of the remaining nine individuals, three used fall stopovers along the eastern coast of Japan and six along coast of the Kamchatka Peninsula, Russia. Two ACP loons did not use a fall stopover location. We identified a spring stopover site for five ACP loons. Three individuals used spring stopover sites along the coast of Japan, one used a spring stopover site along the Kamchatka Peninsula, and the other within the Columbia River Mouth of Washington state.

Loons from the SP used fall stopover sites along the western Alaska coast (Fig 3A). The SP loon with the latest breeding ground departure forwent fall stopover. All other SP loons used at least one fall stopover and one bird used multiple fall stopover sites. Two individuals stopped over in Kuskokwim Bay on the YKD coast and three along the SP where one used a second stopover site along the northern coast of the Alaska Peninsula.

Loons from the YKD had similar fall stopover sites as the SP along the western Alaska coast (Fig 3B). The YKD loon with the latest breeding ground departure forwent fall stopover. All other YKD individuals used at least one fall stopover and more than half used multiple fall stopover sites. All but one loon stayed along the Yukon-Kuskokwim Delta coast for fall stopover where two birds used a second stopover site in Bristol Bay, AK and another near Prince Wales Island, AK.

**Fig 4:**
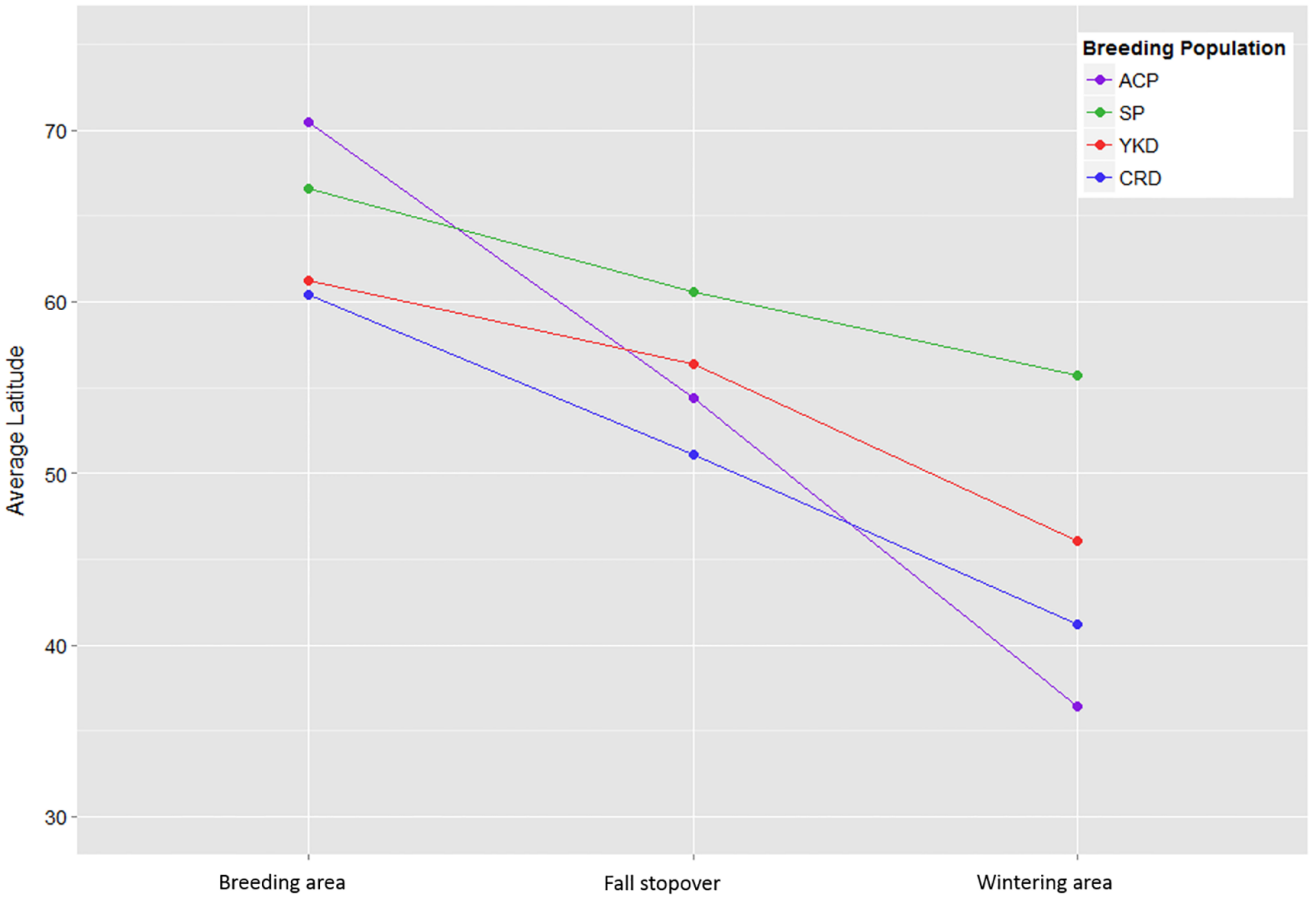
Average latitude of Red-throated Loons at important stages of the annual cycle from 4 different breeding populations. Red-throated Loon breeding populations represent Alaska’s Arctic Coastal Plain (ACP), Seward Peninsula (SP), Yukon-Kuskokwim Delta (YKD), and Copper River Delta (CRD).

Loons from the CRD did not share fall stopover sites with any other population (Fig 3C). All CRD loons used at least one fall stopover and more than half used multiple fall stopover sites. Two individuals stayed within Alaska, one within the Kenai Peninsula and the second in Glacier Bay and Yakutat Bay. Two loons stopped over in the Salish Sea, one in the Columbia River Mouth, one in Grey’s Harbor, Washington and the other near Queen Charlotte Island, Canada. Average fall stopover and wintering site latitudes of SP birds were greater than YKD birds which, in turn, were greater than CRD birds (Fig 4).

### Migration distances and pace

**Fig 5:**
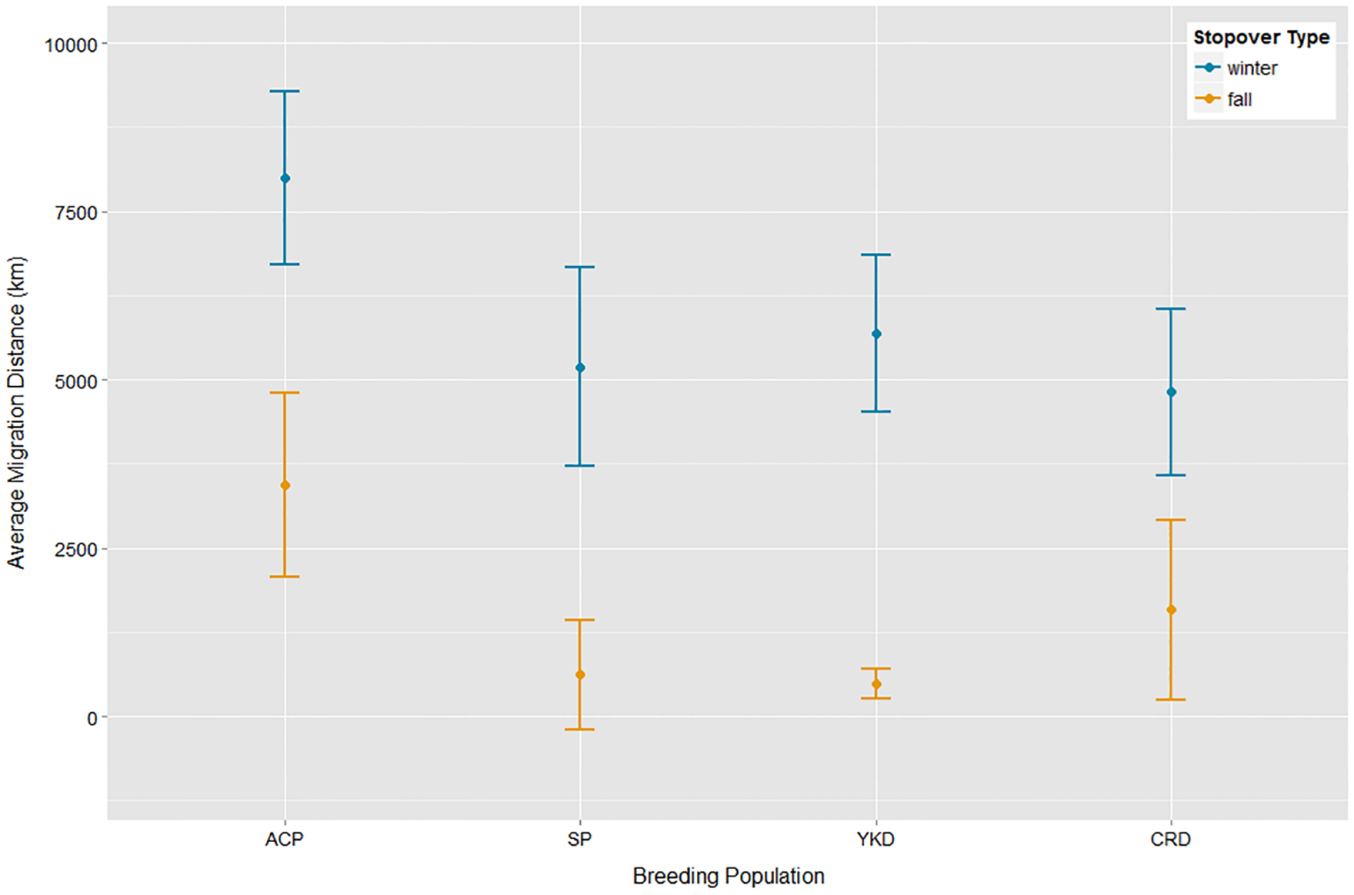
Average distance (km, ± SD) of migrations to fall stopover sites and wintering areas by Red-throated Loons. Red-throated Loon breeding populations represent Alaska’s Arctic Coastal Plain (ACP), Seward Peninsula (SP), Yukon-Kuskokwim Delta (YKD), and Copper River Delta (CRD).

Loons from the ACP traveled the farthest average distance (±SD) to first fall stopover sites (3,908 ± 1,990 km), over twice the distance of CRD loons (1,578 ± 1,344 km) and five times greater than that of SP (614 + 816 km) and YKD loons (489 + 224 km; Fig 5). Likewise for the 24 individuals who reached their respective wintering areas before transmitter failure, ACP loons underwent the longest total average migration to wintering site (7,993 ± 1,285 km, n = 9) generally ~2,000 km farther than the three other breeding populations who traveled similar distances, YKD (5,690 ± 1,165 km), SP (5,189 ± 1,481 km) and CRD (4,816 + 1,224 km). The average pace of migrations from the breeding area to wintering grounds was fastest for ACP loons (73.3 ± 13.7 km/day), followed by SP (68.6 ± 23.0 km/day) and CRD (48.8 ± 12.5 km/day) and YKD (48.0 ± 9.54 km/day).

### Temporal patterns

**Fig 6:**
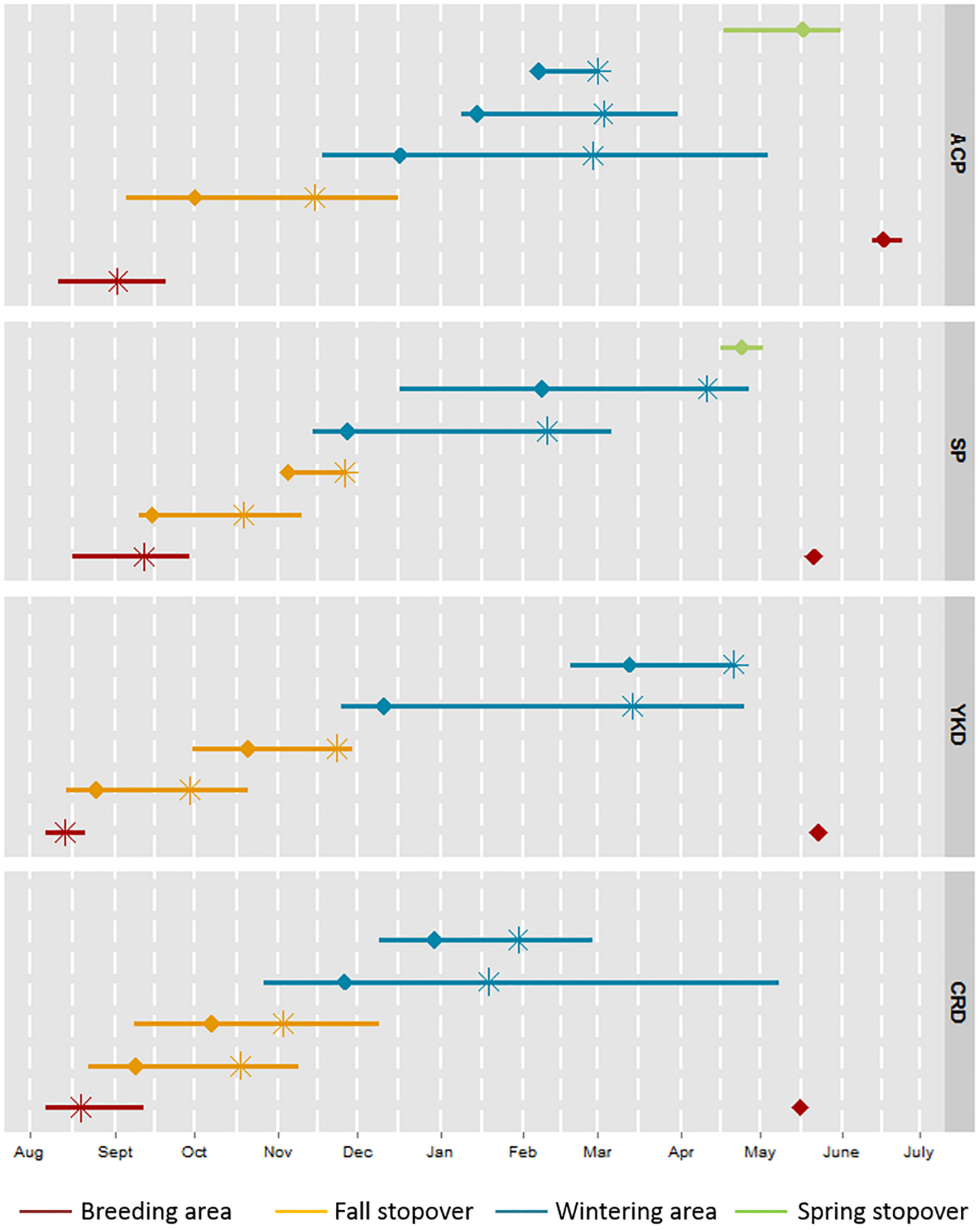
Average temporal migration patterns by Red-throated Loon breeding population. Line length represents date ranges from earliest arrival to latest departure. Average date of arrival denoted by diamond (◊), and average date of departure denoted by asterisk (*). Multiple lines represent different spatially distinct stopover or wintering areas used by loons from a breeding population. Breeding areas represent Alaska’s Arctic Coastal Plain (ACP), Seward Peninsula (SP), Yukon-Kuskokwim Delta (YKD), and Copper River Delta (CRD).

Arrival, departure and duration of stay for stopover and wintering sites as well as in-route migration intervals differed among breeding populations and years (Fig 6). The average day of departure (± SD) from the breeding site was earliest for YKD loons (8/13, ± 5.7) which was a week earlier than CRD (8/19, ± 15.2), three weeks before ACP (9/2, ± 9.2) and a month earlier than SP (9/10, ± 15.2). However, bias in these patterns may exist as breeding success is not known for many of these loons, yet departure time from breeding areas is likely influenced by breeding success, i.e., whether parents are attending young in late summer.

Duration of migration from breeding grounds to first fall stopover increased with distance to fall stopover (Fig 6). ACP loons averaged (± SD) the longest fall migration duration (28 ± 20.1 days) followed by CRD (20 ± 12.5), YKD (13 ± 7.5) and SP (8 ± 9.5). Average arrival date to fall stopover sites was earliest for YKD birds (8/25, range: 8/14–9/12), two weeks earlier than CRD (9/8, range: 8/22–10/13), three weeks before SP (9/15, range: 9/10–9/30) and six weeks earlier than ACP (10/1, range: 9/5–11/8). Average time spent at fall stopover sites was greatest for YKD loons (56.5 ± 17.6 days), followed by CRD (55.6 ± 21.5 days), ACP (44.6 ± 17.9 days), and SP loons (38.2 ± 16.8 days). Average departure date from fall stopover sites was earliest for SP (10/28, range: 10/6–11/26), a little over a week earlier than CRD (11/5, range: 9/16–12/9) and YKD birds (11/7, range: 9/26–11/29) and two weeks earlier than ACP (11/15, range: 10/9–12/16).

Duration of migration from fall stopover sites to wintering grounds increased with distance to wintering grounds. ACP birds averaged the longest migration duration from fall stopover to wintering site (35 ± 14.7 days) followed by YKD (25 ± 24.6), SP (24 ± 16.0) and CRD (16 ± 12.9). Average arrival date to a wintering site was earliest for CRD birds (11/26; range: 10/27–12/20), a day earlier than SP birds (11/27; range: 11/14–12/16), two weeks earlier than YKD birds (12/10; range: 11/25–12/28) and three weeks earlier than ACP (12/17; range: 11/18–1/14). Average time spent at the wintering site was greatest for SP birds (122.0 ± 8.6 days), then YKD birds (107.3 ± 48.5 days), ACP (80.4 ± 47.2), and CRD (71.8 ± 73.5).

## Discussion

### Migratory behavior

We found different patterns of migratory movement among breeding populations of Red-throated Loons in Alaska. For the most northerly breeding population (ACP), loons generally migrated along the western Pacific coastline, largely using the East Asian flyway [42]—a different migration pathway than the three more southerly breeding populations we studied.

For the three other populations, plus one bird from the ACP, all loons traveled down the west coast of North America, leading to some co-occurrence of loons from all populations at similar stopover and wintering sites. Nonetheless, in general, these three populations were geographically ordered in their migration extent and timing. Those wintering farthest north were those from the Seward Peninsula. In contrast, loons breeding at the Copper River Delta, the lowest latitude site, migrated to the southernmost extent. These latitudinal patterns of breeding and wintering locales suggest a chain migration strategy for Red-throated Loons. Chain migration behavior has also recently been identified in Northern Gannets (*Morrus bassanus*), another near-shore piscivorous bird that captures its prey underwater [43].

The difference in general migration patterns between the ACP and all other breeding areas in Alaska is also seen in a diverse suite of circumpolar bird species that migrate between breeding and wintering areas on opposite sides of the Beringian Divide [44–46]. This pattern may be a residual migratory pathway from when the Bering Land Bridge existed in the Bering Sea region and spanned Eurasian and North American continents [47].

### Stopover and wintering area characteristics

Given that loons have high wing loading [48] and travel long distances, stopover and wintering sites likely function as both resting and refueling areas. Additionally, the availability and quality of prey at these sites may be important determinants to individual fitness during the non-breeding season [49–50]. For Red-throated Loons, stopover and wintering areas generally coincided with locations of major coastal upwelling (e.g., California current upwelling) [51–52]. The movement of cool, deep, nutrient rich water to the ocean surface increases levels of primary production [53], creating an abundance of food for seabirds during the non-breeding season. In addition many stopover and wintering sites were located near coastal estuaries, e.g., Fraser River mouth, Puget Sound, San Francisco Bay, which are also typically areas of high productivity [30] important for sub-surface foraging by piscivorous seabirds such as Red-throated Loons [54].

### Pace of migration

Migratory birds can be categorized as those that frequently soar and those that predominantly use flapping flight. Heavy-bodied birds, such as loons, do not soar and thus expend energy in flapping flight and thus may be energetically constrained in how far they can migrate [55]. Nonetheless, Red-throated Loons in this study migrated further than expected, given their body size [55]. Large birds that use flapping flight are also typically constrained to shorter migration distances. Our results suggest that Red-throated Loons do not minimize the duration of migration; instead, they appear to minimize energy expenditure. In this study, Red-throated Loons rarely migrated across land and generally followed coastlines to stopover and wintering areas, minimizing fuel load but increasing overall travel time. Additionally, nearly half of all individuals used multiple stopover and wintering sites during migration and many stopover and wintering sites were subsequent to periods of prolonged migration bursts where individuals remained locally situated for a number of days before migrating again. Lastly, high wing loading may limit the amount of body reserves that Red-throated Loons can store between stopover areas. Multiple stopovers may allow loons to refuel frequently and may be advantageous over longer migration distances.

### Migration distances

The distances traveled between breeding areas and wintering grounds were generally two to four times that of Common Loons [12] and farther than most documented seabird species [2].

The present distribution of Arctic breeding species and differential migration among breeding populations may be attributed to the physical-geographical conditions during the Last Glacial period [47]. All present North American low-arctic animals are postglacial immigrants from the Bering Sea area and current breeding populations generally coincide with ice-free areas during the Last Glacial period [47]. This information also provides insight into present day breeding population locations and differential migration we discovered among Alaska breeding Red-throated Loons. It is presumed that during the Last Glacial period much of the northern coast of the Bering Sea remained ice-bound while the southern coast was virtually ice-free due to warm Pacific Ocean currents [47]. Red-throated Loons depend on marine-derived food during both the breeding and non-breeding seasons, so it is likely that a majority of the population remained along the southern Bering Sea coast year-round. However, as conditions warmed, patterns of both migration and breeding area preference likely changed due to (i) the receding of ice sheets exposing more southern areas (ii) the rise of sea levels submerging wintering and breeding areas within the Bering Sea [47]. This may explain the differential migration we discovered among breeding populations, where ACP maintained the historical migration path across Beringia and eventually continued west as conditions warmed whereas SP, YKD and CRD forwent the western migration and began following a more southerly route as ice began receding southward. Given the differences in migration routes and wintering areas among Red-throated Loon breeding populations, studies of population genetic differentiation within this species could quantify if geographic separation and migratory behaviors have resulted in limited gene flow among breeding populations.

### Conservation concerns

Like many seabirds, Red-throated Loons are a long-lived, low-fecundity species with delayed maturity [11]. These life-history characteristics make population growth rates highly sensitive to small changes in adult mortality. Therefore, understanding differential risks to Red-throated Loons during the non-breeding periods may inform differences observed in population trends among Alaska breeding populations. We found that individuals from the three more southerly breeding populations use similar stopover and wintering areas, indicating an increased risk of species level vulnerabilities. These areas were often found near estuaries and locations of major coastal upwelling and due to their inherent productivity are typically home to other competing bird species as well as high anthropogenic uses areas (e.g., Fraser River Estuary) [56].

Coastal and marine systems are continually inundated with terrestrial, atmospheric and marine derived pollutants [30, 57]. Red-throated Loons are inherently susceptible to marine contaminants as 1) species that undergo long distance migration, and use multiple stopover sites in areas with different environmental standards, and 2) pollutants, particularly lipid-soluble pollutants, bioaccumulate through the food chain affecting high trophic level marine feeders [58]. For example, higher polychlorinated biphenyl (PCB) concentrations were found in ACP breeding birds compared to the other three populations [21]. These results strongly suggest that differences in contaminant profiles among populations is linked to contrasting migration strategies as the ACP is the only breeding population to migrate westward to East Asia. Preliminary satellite telemetry data in Yellow-billed Loons found a similar pattern in that populations breeding on the ACP and migrating to East Asia had higher rates of mercury exposure than breeding populations in Canada that winter in North America [16–17].

### Conclusions

To our knowledge this is the first systematic effort to describe the migration patterns of Red-throated Loons. Results from this study highlight migration routes and important areas used for stopovers and winter by four different breeding populations. These shared pathways and locations by multiple breeding populations highlight key areas and flyways to aid in future conservation efforts. Since populations of Red-throated Loons breeding on the ACP continue to decline and this was the only breeding population to migrate outside of North America, future research should focus on this population, especially with the potential for contaminant exposure from sources in East Asia. Efforts should be made to expand satellite transmitter deployment to other Red-throated Loon breeding populations (e.g., Western Canada and Russia) since their breeding range is circumpolar and our efforts were restricted to populations breeding in Alaska. Red-throated Loons are also the only loon species to complete a simultaneous remigial molt in the fall [11] and identifying molting areas is important as this may be a vulnerable period of the annual cycle. Habitat analyses of core staging and wintering areas could also aid in determining potential impacts to loon populations.

## Supporting information

S1 Table Summary of satellite platform terminal transmitter (PTT) data for each individual Red-throated Loon from four Alaskan breeding populations. (TIF)

S2 Table Summary of stopover locations, and wintering sites for individual Red-throated Loons from four Alaskan breeding populations. (TIF)
