# Supplementary figures and images for: International migration patterns of Red-throated Loons (Gavia stellata) from four breeding populations in Alaska
Source: PLoS One. 2018 Jan 10;13(1):e0189954. doi: 10.1371/journal.pone.0189954 (PMC5761837; doi:10.1371/journal.pone.0189954)

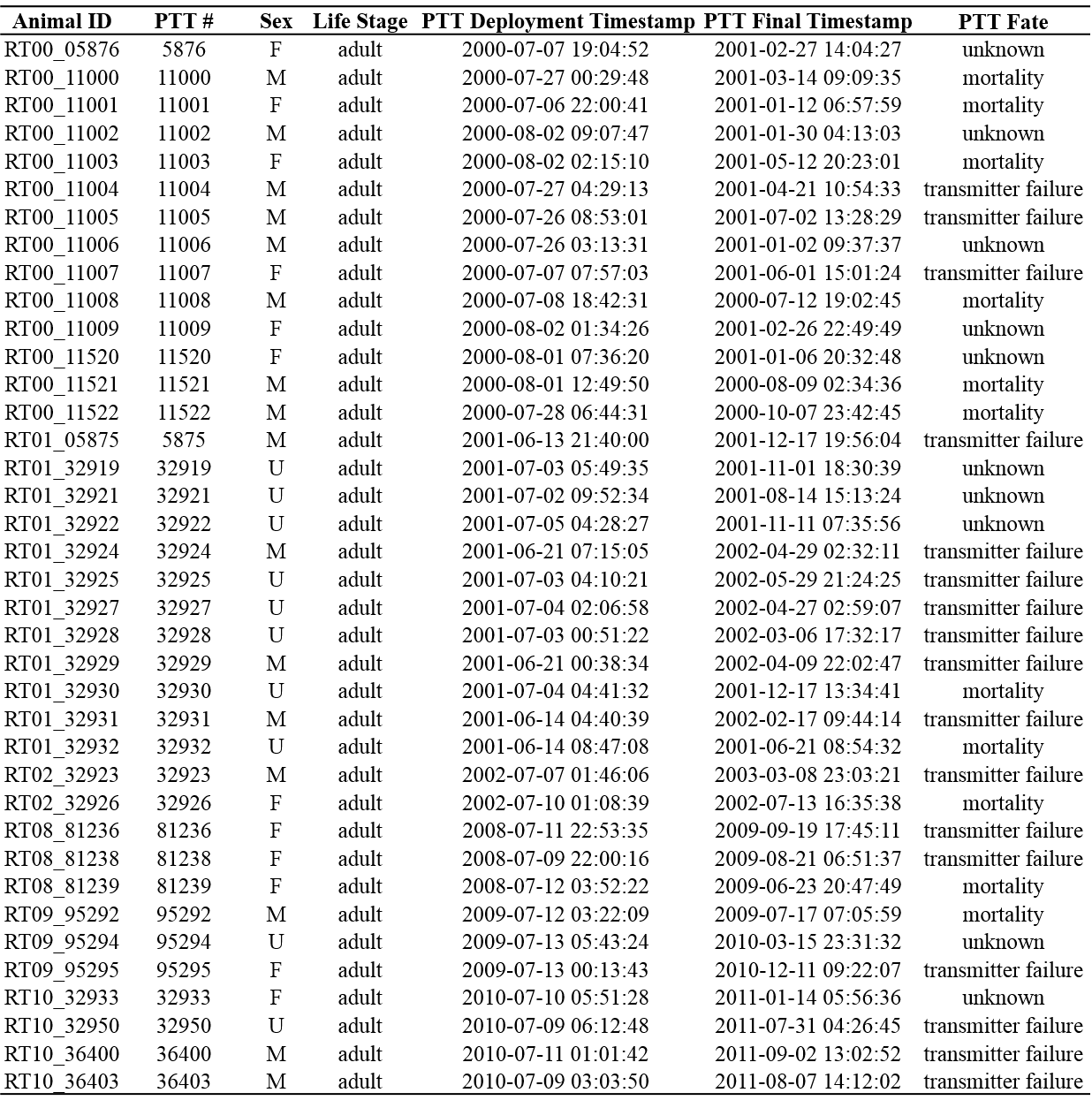

Supplement: S1 Table — (TIF) [file pone.0189954.s001.tif]

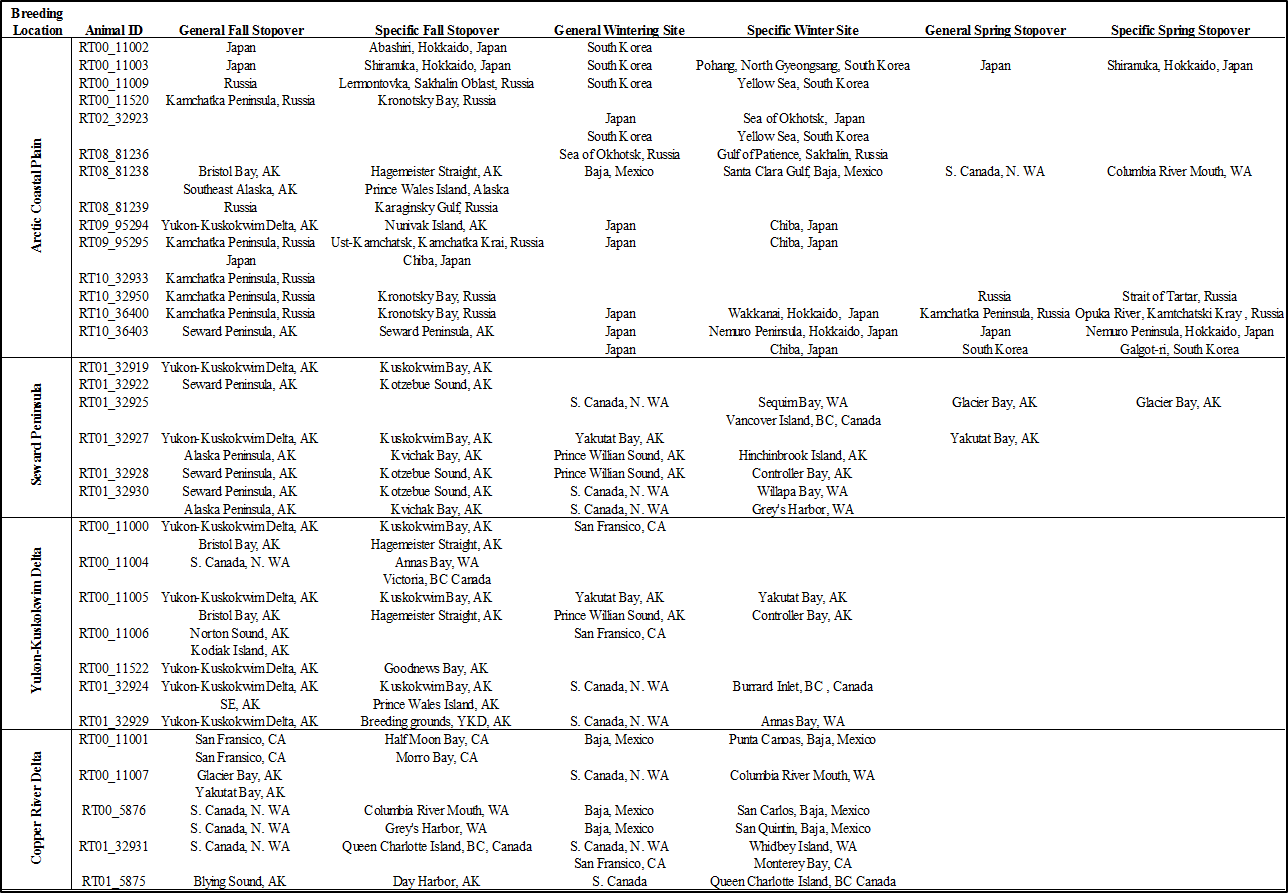

Supplement: S2 Table — (TIF) [file pone.0189954.s002.tif]
